# Clinical Evaluation of a Combination of Non‐Invasive Technologies to Improve the Effectiveness of Postpartum Abdominal Treatment

**DOI:** 10.1111/jocd.70330

**Published:** 2025-07-10

**Authors:** Hannah Lo Fui Jun, Tingsong Lim, Grace Tay Wen Yue, Chua Chong Hwee, Tee Xiang Jie, Gwen Ong, Yap Ching Shi, Ding Siew Wen, Lim Sook Wah

**Affiliations:** ^1^ Clique Clinic Bangsar Clique Clinic Kuala Lumpur Malaysia; ^2^ Clique Clinic Petaling Jaya Clique Clinic Petaling Jaya Selangor Malaysia; ^3^ Gloria Confinement Centre George Town Pulau Pinang Malaysia

**Keywords:** postpartum abdomen, radiofrequency, skin laxity, stretch marks, targeted pressure energy

## Abstract

**Background:**

Pregnancy induces a range of physical changes in the female body, some of which may persist long after delivery. This can result in body dissatisfaction and reduced quality of life. Skin laxity, diastasis recti, and stretch marks pose a challenge to achieving the desired appearance.

**Aim:**

This study aims to investigate the potential benefits of the consecutive use of Radiofrequency (RF) + Targeted Pressure Energy (TPE) and HIFEM + RF for the treatment of postpartum abdomen and improvement of appearance.

**Methods:**

This prospective study enrolled 38 postpartum participants for the consecutive treatments of Targeted Pressure Energy plus radiofrequency and HIFEM plus radiofrequency on their abdomen. Follow‐up visits were scheduled at 3 months and 12 months. Global Aesthetic Improvement Scale (GAIS) evaluation, weight measurements, and circumference measurements were performed at 3 levels corresponding with 5 cm above the navel (circumference A), at navel level (circumference B), and 5 cm below the navel (circumference C).

**Results:**

The study results show a reduction in circumference. At 3 months post‐treatment, circumference A decreased by 1.57 cm, circumference B by 2.09 cm, and circumference C by 1.83 cm. At 12 months post‐treatment, circumference A decreased by 3.16 cm, circumference B by 4.69 cm, and circumference C by 4.06 cm. The GAIS evaluation at 3 months post‐treatment showed average improvements of 1.53 points for stretch marks, 1.58 points for skin laxity, 1.77 points for muscle toning, and 1.64 points for slimming effect. At 12 months post‐treatment, the GAIS evaluation showed improvements of 1.6 points for stretch marks, 1.8 points for skin laxity, 1.9 points for muscle toning, and 1.9 points for slimming effect.

**Conclusion:**

The consecutive use of RF + TPE and HIFEM + RF has shown to be effective in the treatment of postpartum abdomen and has resulted in desirable effects. Overall appearance has improved significantly, which may also indicate an increased quality of life. The study results show that both devices complement each other, and their combined use leads to better outcomes than standalone therapy, particularly in terms of enhanced results in body contouring and skin tightening.

**Abbreviations**
GAIS: Global Aesthetic Improvement Scale
RF: Radiofrequency
TPE: Targeted Pressure Energy

## 1 Introduction

During pregnancy, women undergo significant physical changes, including a 1.5‐fold increase in intra‐abdominal volume [1], which places considerable strain on their bodies. The skin stretches extensively and has been shown to lose elasticity, a condition observed to persist even 4 months after delivery, resulting in residual skin laxity. This stretching also increases the risk of developing stretch marks, a form of chronic dermal scarring. In Asian skin, excessive fibroblast during wound healing leads to thicker collagen deposition and an increased risk of visible scarring and post‐inflammatory hyperpigmentation [2]. Combined with diastasis recti and weight retention, these factors contribute to a loss of waist definition and present a cosmetic challenge.

These changes are common during and after pregnancy, and lack effective prevention [3], and are often resistant to lifestyle‐based interventions. Women often feel societal pressure to return to their pre‐pregnancy bodies and maintain a slim figure with unmarked skin [4]. Moreover, women from Asian cultures may face additional obstacles related to traditions that can affect postpartum weight management, such as restrictions on physical activity after childbirth [5, 6].

RF has been widely used in cosmetic dermatology as a non‐invasive method for skin rejuvenation [7]. The radiofrequency energy absorption and transformation to heat cause a release of various growth factors, such as fibroblast growth factor 2 [8], transforming growth factor beta 1, as well as dermal fibroblast stimulation, leading to the upturned collagen and elastin production. The old collagen and elastin fibers, moreover, undergo a reorganization, leading to an improved structure, similar to younger fibers. Heating of the fat tissue in the temperature range of 43°C–45°C, on the other hand, enforces adipocytes to undergo apoptosis, resulting in their elimination, without the risk of inflammation or necrosis [9].

The radiofrequency effect is enhanced when coupled with other modalities. RF, when paired with TPE, has been shown to effectively treat skin laxity and cellulite by increasing dermal thickness and remodeling adipose interlobular septae, with enhanced collagen and elastin content [10]. The benefits of TPE rely on the proliferative activity of fibroblasts and the creation of suitable conditions for collagen re‐synthesis. The combination of RF and TPE induces significant changes in the connective and subcutaneous tissues and is suggested to be more effective than using either RF or TPE alone or consecutively [11, 12].

The simultaneous application of HIFEM technology became an innovative method among non‐invasive body contouring options. While RF targets skin laxity and fat tissue, HIFEM technology is effective for muscle strengthening [13]. The electromagnetic field of HIFEM induces supramaximal contractions in the treated muscle, akin to intense physical activity, leading to muscle hypertrophy and hyperplasia through the activation of satellite cells and heat‐shock proteins [14, 15]. The combination with RF enhances the HIFEM effect by increasing blood flow, which improves nutrient delivery and waste product drainage [16]. This, in turn, boosts the results of abdominal body shaping, as the simultaneous application of RF and HIFEM leads to a significant increase in rectus abdominis muscle thickness and a reduction in subcutaneous fat. Together, these technologies amplify both muscle growth and fat reduction, optimizing the overall body contouring effect [13].

This study aims to investigate the potential benefits of the consecutive use of RF + TPE and HIFEM + RF for improving the appearance of the postpartum abdomen.

## 2 Methods

This prospective, open‐label, single‐center interventional study enrolled thirty‐eight postpartum women (*n* = 38, mean age 35.2 ± 3.1 years, BMI of 21.6 ± 2.8 kg/m [2]), who underwent abdominal treatment consisting of consecutive sessions of RF + TPE and HIFEM + RF.

Among the inclusion criteria were a postpartum period of at least three months for patients with vaginal delivery, and at least 6 months postpartum period for patients delivering through C‐section, but no longer than 36 months post‐delivery, and willingness to have photographs of their abdomen taken. The exclusion criteria were the presence of the intrauterine device, pregnancy, nursing, or any other medical conditions that contraindicate the application of electromagnetic field, radiofrequency, and targeted pressure energy, such as cardiovascular disease, malignant tumor, or electronic implants.

Subjects were given instructions about the study procedures, voluntarily participated in the study, and signed a written informed consent. The study adhered to the Declaration of Helsinki principles.

Study participants received six treatments, spaced 10 days apart. For the first four procedures, they underwent 30 min of HIFEM + Synchronized Radiofrequency (Emsculpt Neo, BTL Industries Inc.), followed by 15 min of Targeted Pressure Energy + Radiofrequency (Emtone, BTL Industries Inc.). A 15‐minute break was provided between treatments to allow the area to cool down. The subjects received only a 30‐minute HIFEM + Synchronized Radiofrequency therapy for the last two scheduled procedures. The intensities of each modality were adjusted to maximally tolerated levels according to the subjects' feedback. Follow‐up visits were scheduled 3 months and 12 months after the last treatment.

Circumference measurements and standardized photographs were taken at baseline, 3 months, and 12 months post‐treatment. Circumference was measured at three levels: at the umbilical level (B), 5 cm above (A), and 5 cm below the umbilical level (C). The umbilical circumference was measured from the umbilicus to the floor and marked on the body to ensure measurement accuracy.

Subject photographs were taken from two angles, from the front and from the side. Three independent clinicians (an aesthetic doctor, a body specialist, and a specialist in postpartum care) evaluated the obtained photographs according to the GAIS, where 1 means worse than the initial condition, and 2 means drastic improvement, easily noticeable.

The patients' height was measured only at the baseline visit, while weight was measured at the baseline and both follow‐up visits to track changes and calculate BMI.

Descriptive analytics (mean value, standard deviation) were performed. The Shapiro–Wilk normality test was used to verify whether the variables were normally distributed. The statistical significance was assessed using the Wilcoxon matched‐pairs test, with α = 0.05 considered the significance level.

## 3 Results

Thirty‐eight subjects (*n* = 38, females, 35.2 ± 3.1 years, BMI 21.5 ± 2.8) were enrolled in this study. All subjects attended their 3‐month follow‐up visits, and sixteen (*n* = 16, females, 35.4 ± 3.2 years, BMI 21.7 ± 2.4) willingly participated in the 12‐month follow‐up visit.

The subjects (*n* = 38) had on average 2.1 ± 0.9 successful pregnancies in the past, with six subjects having twins in their last pregnancy prior to the start of the study. The mean postpartum period was 12.2 ± 9.4 months. Twenty‐four subjects had a natural delivery, while fourteen subjects underwent a C‐section.

Changes in all three measured circumferences were statistically significant with *p* < 0.0001. At the 3‐month post‐treatment visit, the maximum reductions observed were 6 cm, 10 cm, and 7 cm for circumferences A, B, and C, respectively. Women with three or more pregnancies exhibited greater reductions in circumferences B and C, while the reduction in circumference A was similar between women with three or more pregnancies and those with two or fewer.

**FIGURE 1:**
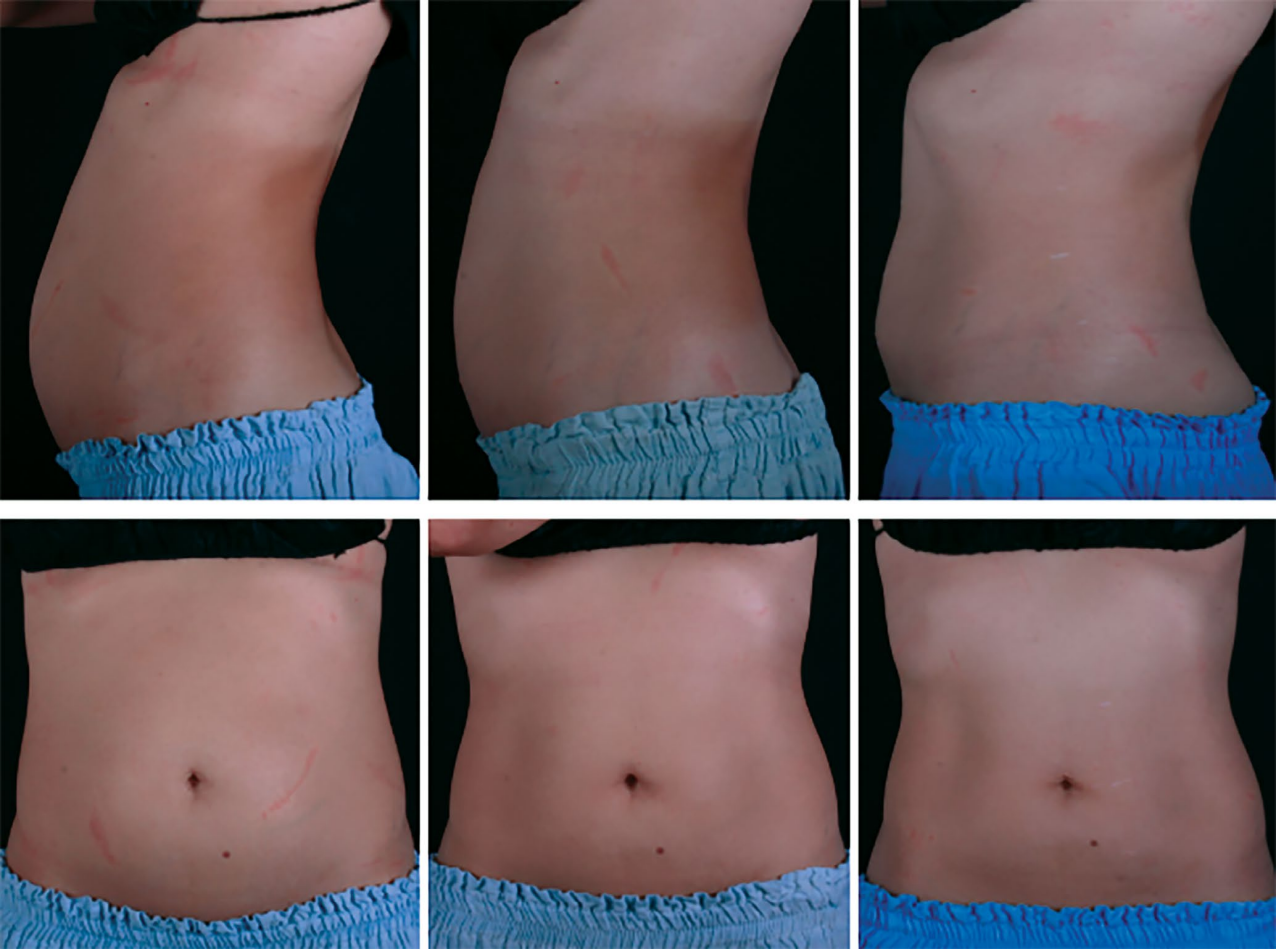
Baseline (left), 3 months post‐treatment (middle), 12 months post‐treatment (right). Visible slimming and muscle toning effect on a 39‐year‐old subject, 8 months postpartum. At 12 months post‐treatment, the patient's circumference decreased by 10.0 cm (A: 5 cm above umbilicus), 13.0 cm (B: at umbilicus), and 11.0 cm (C: 5 cm below umbilicus).

**FIGURE 2:**
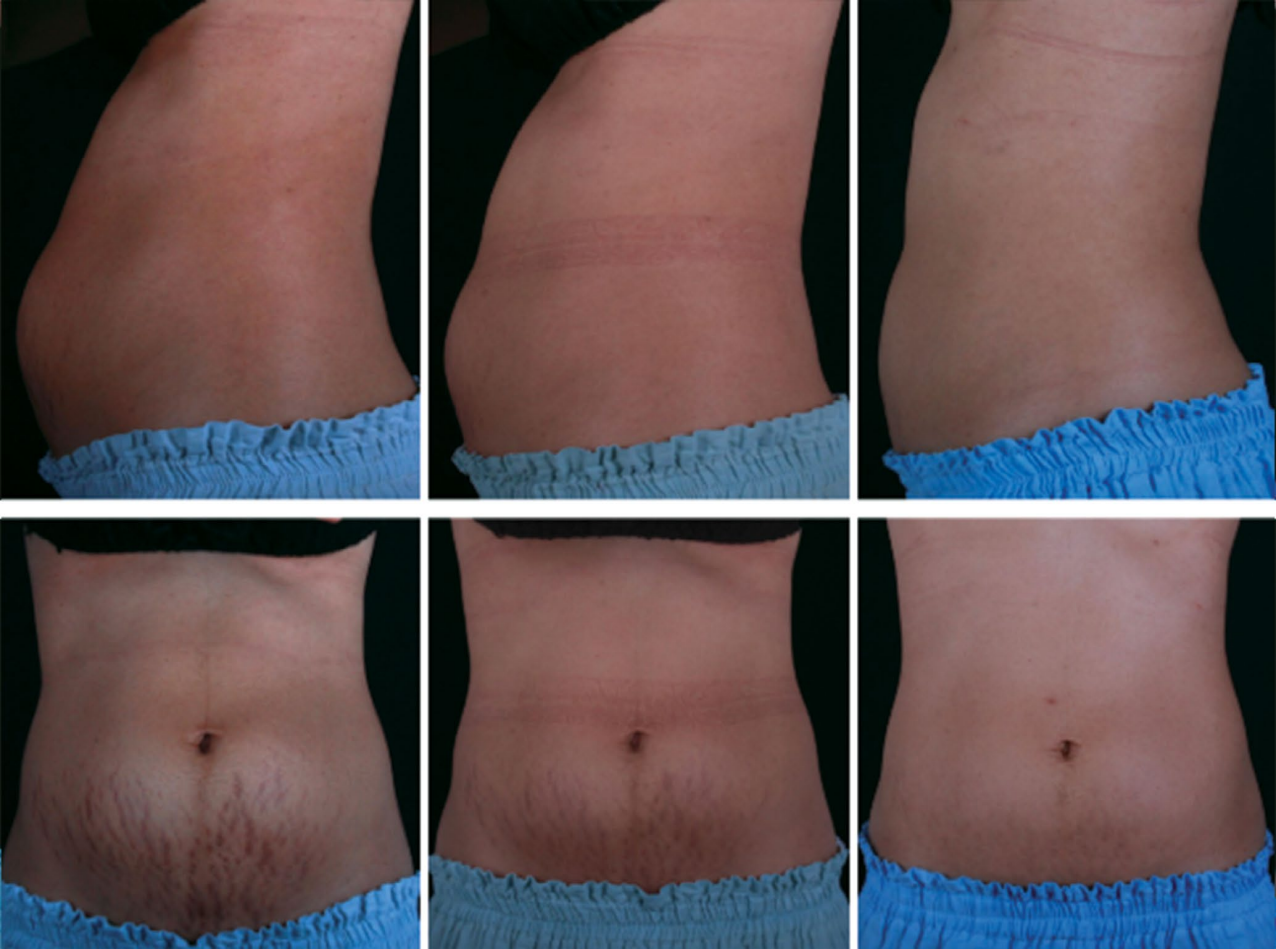
Baseline (left), 3‐month post‐treatment (middle), 12 months post‐treatment (right). A noticeable reduction of stretch mark appearance, as well as a slimming effect and muscle toning, on a 32‐year‐old 3 months postpartum patient. At 12 months post‐treatment, the patient's circumference decreased by 1.5 cm (A: 5 cm above umbilicus), 2.0 cm (B: at umbilicus), and 1.0 cm (C: 5 cm below umbilicus).

**FIGURE 3:**
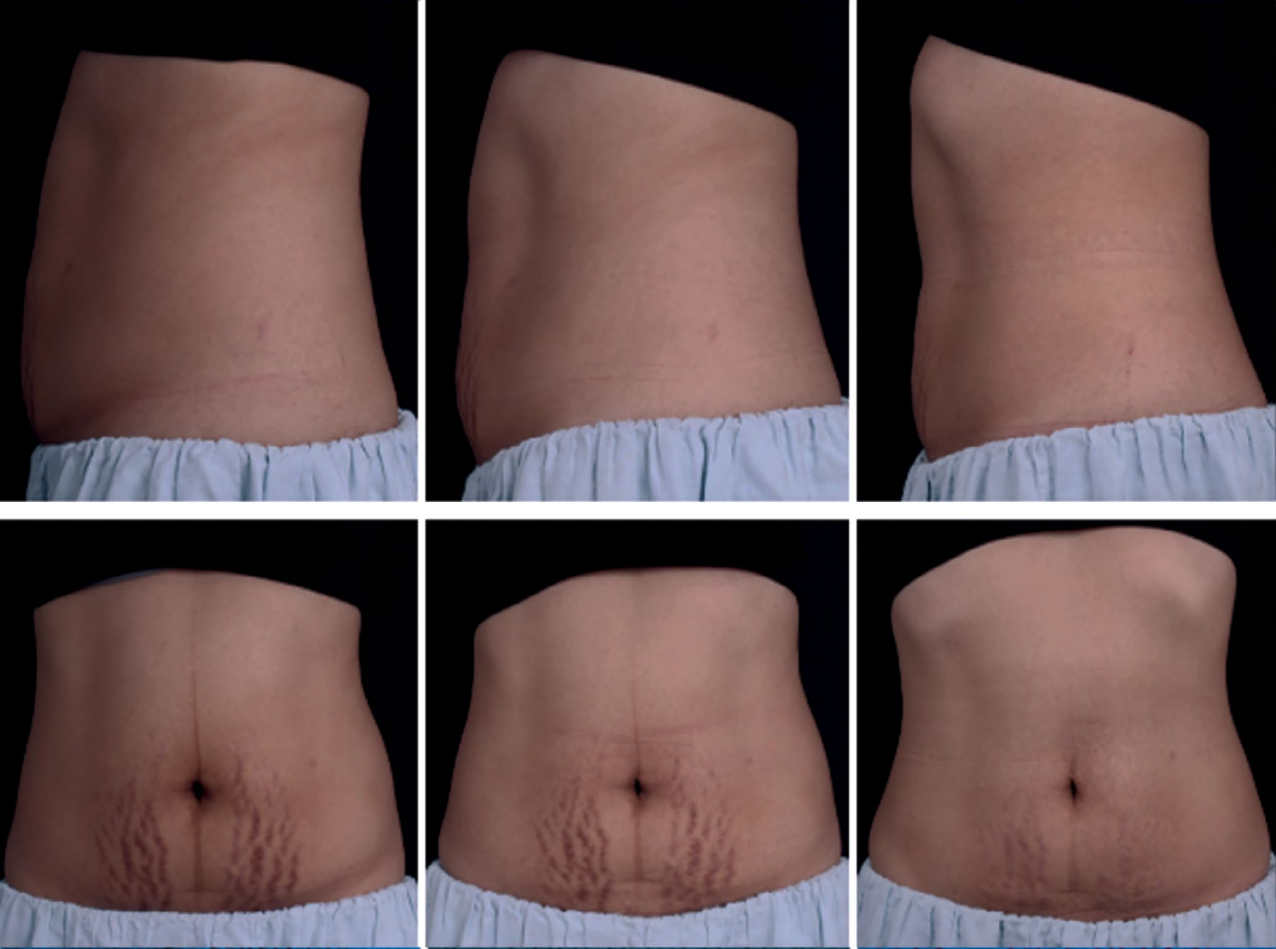
Baseline (left), 3‐month post‐treatment (middle), 12 months post‐treatment (right). Improvement in the appearance of stretch marks and muscle toning on a 30‐year‐old 3 months postpartum patient. The patient's circumference at 12 months post‐treatment decreased by 2.0 cm (A: 5 cm above umbilicus), 4.0 cm (B: at umbilicus), and 4.0 cm (C: 5 cm below umbilicus).

At the 12‐month post‐treatment visit, none of the subjects experienced an increase in circumference, and all 16 subjects showed a reduction. One subject showed maximum reductions across all three measurements: 10 cm for circumference A, 13 cm for circumference B, and 11 cm for circumference C. Women with two or fewer pregnancies experienced an above‐average reduction in circumference, resulting in a greater decrease than that observed in women with three or more pregnancies. Women who had C‐sections showed greater reductions in circumferences A and B compared to those with natural births. See the summary of key results in Tables 1 and 2, and visual documentation of treatment effects in selected participants at key timepoints is available in Figures 1, 2, and 3. There were no adverse events or side effects reported throughout the study.

**TABLE 1:** Summary of GAIS scores at 3 M and 12 M follow‐up.

Measure	3 months (*n* = 38)	12 months (*n* = 16)
GAIS‐Stretch Marks	1.5 ± 0.6	1.6 ± 0.4
GAIS‐Skin Laxity	1.6 ± 0.6	1.8 ± 0.2
GAIS‐Slimming Effect	1.6 ± 0.4	1.9 ± 0.1
GAIS‐Muscle Toning	1.8 ± 0.3	1.9 ± 0.1

**TABLE 2:** Reduction in circumference measurements at 3 M and 12 M follow‐up.

Measure	3 months (*n* = 38)	12 months (*n* = 16)
Circumference A	1.57 ± 1.44 cm	3.16 ± 2.81 cm
Circumference B	2.09 ± 2.21 cm	4.69 ± 4.06 cm
Circumference C	1.83 ± 1.99 cm	4.06 ± 3.02 cm

## 4 Discussion

This clinical study aimed to explore the potential benefits of consecutive treatments using HIFEM+RF and RF + TPE technologies for postpartum abdominal improvement. The results demonstrated measurable improvements in abdominal circumference and overall appearance. At 3 months post‐treatment, the average reductions were 1.57 cm, 2.09 cm, and 1.83 cm in the abdominal circumference, measured 5 cm above the umbilical level, at the umbilical level, and 5 cm below the umbilical level, respectively. Aesthetic outcomes evaluated using the GAIS scale showed improvements in stretch marks (1.5 points), skin laxity (1.6 points), muscle toning (1.8 points), and a slimmer abdomen (1.6 points). At 12 months, GAIS scores remained high, with 1.6 points for stretch marks, 1.8 points for skin laxity, and 1.9 points for muscle toning and slimming effect. These findings support the effectiveness of combining both technologies within a single session to address multiple postpartum concerns.

The suggested mechanism of improved appearance is increased collagen and elastic fiber content in the dermis. RF energy stimulates neocollagenesis and neoelastogenesis by heating the dermis and subcutaneous tissue, which helps improve skin laxity and reduce stretch marks [17]. It also promotes blood flow and induces adipocyte apoptosis, contributing to localized fat reduction. TPE improves skin elasticity as it induces lipolysis and neovascularization and increases collagen fiber density [11]. HIFEM induces an electric current in the underlying tissues, causing involuntary supramaximal muscle contractions. Stimulation of the muscles promotes muscle tissue hypertrophy, also known as muscle building [18, 19]. The combined use of these modalities enables comprehensive targeting of skin, fat, and muscle layers, which results in more effective contouring of the abdominal area [12, 20, 21, 22, 23, 24].

Compared to other non‐invasive treatments like standalone radiofrequency, cryolipolysis, and ultrasound, which often cause temporary swelling and delayed results, the absence of side effects with HIFEM+RF and RF + TPE is a notable advantage [18].

Beyond physical outcomes, these treatments may also support psychological well‐being. The postpartum period often involves significant physical and emotional challenges, with body dissatisfaction contributing to stress and, in some cases, postpartum depression [25]. Aesthetic improvements may help alleviate appearance‐related stress and contribute to improved quality of life during this period. The convenience and safety of these noninvasive procedures make them particularly appealing for new mothers managing demanding routines [26].

The main strength of this study is the long follow‐up, which was conducted for up to 12 months. Another study strength is the number of enrolled patients, with 38 attending the 3‐month follow‐up visit. Despite a reduced number at the 12‐month follow‐up (*n* = 16), the sample size remained adequate for meaningful analysis. Methodological limitations include the absence of a placebo group and a group receiving treatment with only one of the two technologies. Future research would benefit from increasing the number of subjects evaluated at the 12‐month post‐treatment visit and extending the follow‐up observation. In addition, it would be beneficial to perform an MRI and ultrasound evaluation of the results. Nevertheless, more attention could be focused on pairing the two technologies, which could lead to the development of new devices utilizing the benefits of HIFEM+RF and RF + TPE.

## 5 Conclusions

Our study shows that consecutive noninvasive treatments by HIFEM + Synchronized RF and RF + TPE significantly improve the abdominal appearance of postpartum women. The treatments reduce stretch marks, tighten skin, and enhance muscle tone. No adverse effects were reported, further supporting the treatment's safety and efficacy for post‐pregnancy abdominal concerns.

## Author Contributions

All authors contributed equally.

## Ethics Statement

The authors confirm that the ethical policies of the journal, as noted on the journal's author guidelines page, have been adhered to. All subjects voluntarily participated and signed a written informed consent. Additionally, informed consent was obtained from all participants for the use of their photographs.

## Conflicts of Interest

The authors declare no conflicts of interest.

## Data Availability

The data that support the findings of this study are available on request from the corresponding author. The data are not publicly available due to privacy or ethical restrictions.
